# Supplementary material for: Public perspectives and media reporting of wolf reintroduction in Colorado
Source: PeerJ. 2020 May 7;8:e9074. doi: 10.7717/peerj.9074 (PMC7224228; doi:10.7717/peerj.9074)
Supplement: Supplemental Information 2 [file peerj-08-9074-s002.docx]

**Codebook for “Public perspectives and media reporting of wolf reintroduction in Colorado” by Niemiec et al. (2020)**

| Variable (Name) | Values | Type |
| --- | --- | --- |
| Assigned participant identifier (participantID) | 1-734 | Nominal |
| Extent of perceived direct impact on livelihood or quality of life (impactExtent) | 1: Strong negative impact  2: Moderate negative impact  3: Slight negative impact  4: No impact  5: Slight positive impact  6: Moderate positive impact  7: Strong positive impact | Ordinal (Likert) |
| Description of negative impacts (impactsNegative) | Open response | Nominal |
| Description of positive impacts (impactsPositive) | Open response | Nominal |
| Acceptability of limiting the number of wolves if they cause declines in deer and elk populations in certain areas (mgmtLimitDeerElk) | 1: Highly unacceptable  2: Moderately unacceptable  3: Slightly unacceptable  4: Neither  5: Slightly acceptable  6: Moderately acceptable  7: Highly acceptable | Ordinal (Likert) |
| Acceptability of capturing and lethally removing a wolf if it is known to have caused loss of livestock (mgmtLethalRemoval) | 1: Highly unacceptable  2: Moderately unacceptable  3: Slightly unacceptable  4: Neither  5: Slightly acceptable  6: Moderately acceptable  7: Highly acceptable | Ordinal (Likert) |
| Acceptability of compensating landowners for loss of livestock caused by a wolf (mgmtCompLivestock) | 1: Highly unacceptable  2: Moderately unacceptable  3: Slightly unacceptable  4: Neither  5: Slightly acceptable  6: Moderately acceptable  7: Highly acceptable | Ordinal (Likert) |
| Acceptability of using a portion of state hunting and fishing license dollar to compensate landowners for loss of livestock caused by a wolf (mgmtCompLivestockLicense) | 1: Highly unacceptable  2: Moderately unacceptable  3: Slightly unacceptable  4: Neither  5: Slightly acceptable  6: Moderately acceptable  7: Highly acceptable | Ordinal (Likert) |
| Acceptability of using a portion of state tax dollars to compensate landowners for loss of livestock caused by a wolf (mgmtCompLivestockTax) | 1: Highly unacceptable  2: Moderately unacceptable  3: Slightly unacceptable  4: Neither  5: Slightly acceptable  6: Moderately acceptable  7: Highly acceptable | Ordinal (Likert) |
| Acceptability of allowing a recreational hunt of wolves once they have reached a certain population size that exceeds recovery goals (mgmtRecHunt) | 1: Highly unacceptable  2: Moderately unacceptable  3: Slightly unacceptable  4: Neither  5: Slightly acceptable  6: Moderately acceptable  7: Highly acceptable | Ordinal (Likert) |
| Intention to vote on reintroducing the gray wolf to Colorado given opportunity (votingIntention) | 0: Vote against reintroduction  1: Vote for reintroduction | Binary |
| Extent of identification as a wildlife advocate (identifyWildAdv) | 1: Do not identify at all  2: Identify slight amount  3: Identify moderate amount  4: Identify a great deal | Ordinal (Likert) |
| Extent of identification as an animal rights advocate (identifyAnimRightsAdv) | 1: Do not identify at all  2: Identify slight amount  3: Identify moderate amount  4: Identify a great deal | Ordinal (Likert) |
| Extent of identification as a gun rights advocate (identifyGunRightsAdv) | 1: Do not identify at all  2: Identify slight amount  3: Identify moderate amount  4: Identify a great deal | Ordinal (Likert) |
| Extent of identification as a property rights advocate (identifyPropRightsAdv) | 1: Do not identify at all  2: Identify slight amount  3: Identify moderate amount  4: Identify a great deal | Ordinal (Likert) |
| Extent of identification as a hunter (identifyHunter) | 1: Do not identify at all  2: Identify slight amount  3: Identify moderate amount  4: Identify a great deal | Ordinal (Likert) |
| Extent of identification as a rancher (identifyRancher) | 1: Do not identify at all  2: Identify slight amount  3: Identify moderate amount  4: Identify a great deal | Ordinal (Likert) |
| Extent of identification as a conservationist (identifyConserv) | 1: Do not identify at all  2: Identify slight amount  3: Identify moderate amount  4: Identify a great deal | Ordinal (Likert) |
| Gender identification (gender) | 1: Male  2: Female  3: Non-binary/third gender/prefer to self-describe | Nominal |
| Number of people under 18 in household (nChildren) | 0-10 | Integer |
| Pets in household (pets) | 1: Dog(s)  2: Cat(s)  3: Other(s)  4: No pets  (Multiple response) | Nominal |
| Types of other pets in household (petsOther) | Open response | Nominal |
| Annual household income level before taxes (income) | 1: Less than $10,000  2: $10,000 to less than $25,000  3: $25,000 to less than $50,000  4: $50,000 to less than $100,000  5: $100,000 to less than $250,000  6: $250,000 or more | Ordinal |
| Highlest level of education completed (education) | 1: Less than high school  2: High school diploma or equivalent (e.g, GED)  3: 2-year associate’s degree or trade school  4: 4-year college degree  5: Advanced degree beyond 4-year college degree | Ordinal |
| Size of current residence or community (commSize) | 1: Large city with 250,000 or more people  2: City with 100,000 to 249,999 people  3: City with 50,000 to 99,999 people  4: Small city with 25,000 to 49,999 people  5: Town with 10,000 to 24,999 people  6: Town with 5,000 to 9,999 people  7: Small town or village with less than 5,000 people  8: A farm or rural area | Ordinal |
| Age based on year of birth (age) | 18-89 | Integer |
| Binned age group (ageGroup) | 18-34, 35-54, 55+ | Nominal |
| Sampling region of Colorado (region) | Western Slope, Front Range, Eastern Plains | Nominal |
| Sampling weight (weight) | 0.37, 1.66, 0.26 | Discrete |
