# Supplementary material for: Public perspectives and media reporting of wolf reintroduction in Colorado
Source: PeerJ. 2020 May 7;8:e9074. doi: 10.7717/peerj.9074 (PMC7224228; doi:10.7717/peerj.9074)
Supplement: Supplemental Information 4 [file peerj-08-9074-s004.docx]

**Supplementary Materials**

***Weighting of Survey Data***

Weighting factors are applied to a statistical sample in order to assign data points lighter, or heavier, importance as a way to ensure that a sample is representative and accurately reflects the target population. We weighted our data by geography to ensure our sample represented the overall population distribution across different Colorado regions. The weighting methods used here are those detailed in *Survey Research and Analysis: Application in Parks, Recreation and Human Dimension* (Vaske, 2008). Our sample included 365 responses from the Front Range, 277 from the Western Slope, and 92 from the Eastern Plains. Through our weighting procedure, we made our sample representative of the following population distribution obtained through the American Community Survey (2017): 82.56% of the population in the Front Range, 14.12% of the population in the Western Slope, and 3.31% of the population in the Eastern Plains. The weighting factor per region was calculated as the ACS 2017 population % / sample %. We calculated the weighting factor per region with the following formulas: Front Rage x = 82.56431 / 49.72752 = 1.66033436; Western Slope x = 14.12197 / 37.73842 = 0.37420679; and, Eastern Plains x = 3.3137 / 12.53406 = 0.26437691. See Table S-1 and S-2 below for the numbers used in the weighting factor.

***Additional Figures and Tables***


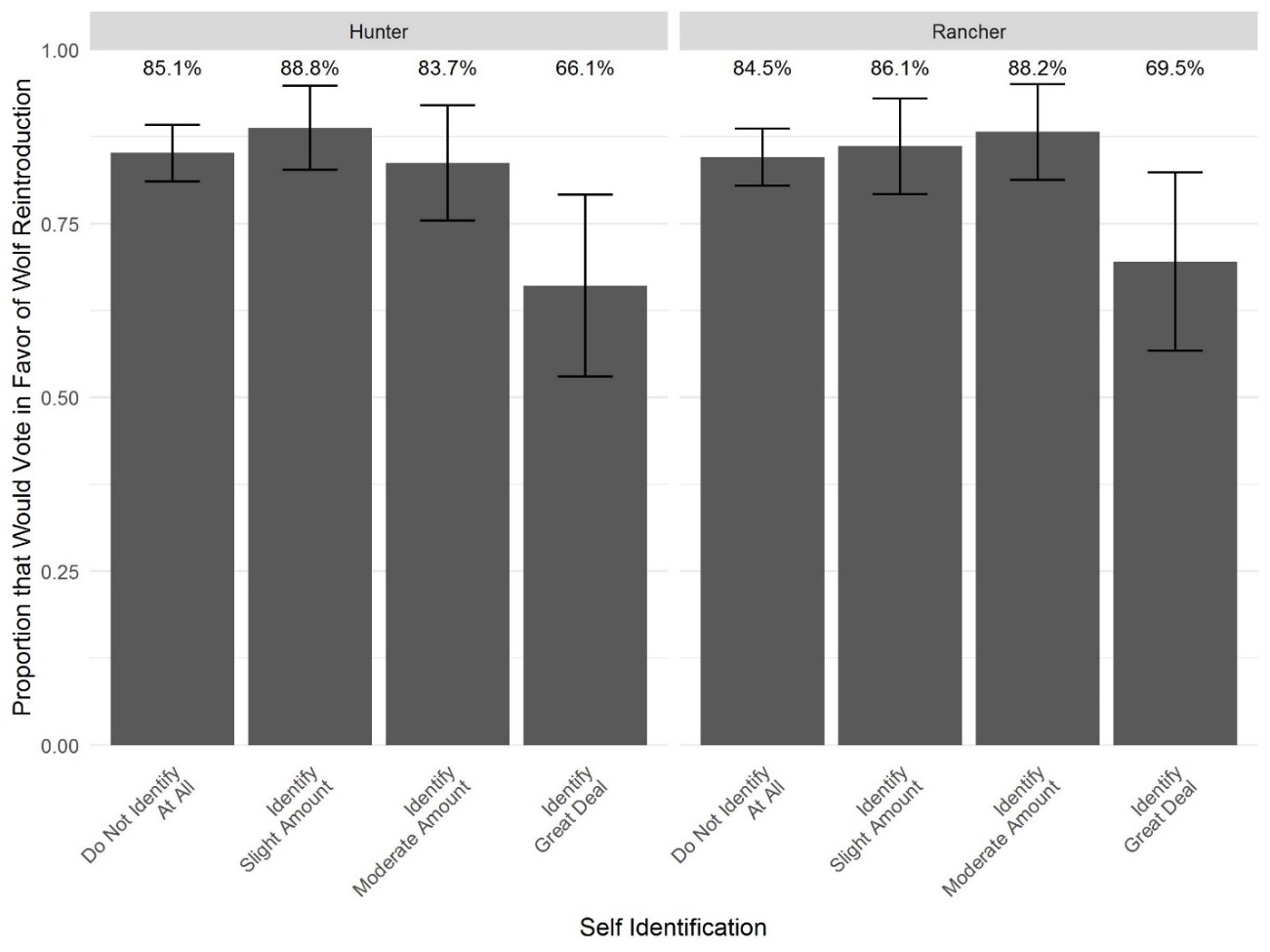


**Figure S-1:** Voting intention by degree of self-identification among hunters and ranchers. The categories “Identify Slight Amount” through “Identify Great Deal” are binned as “Identify As” in Figure 4. Bars depict the proportion of each group in favor of reintroduction (weighted by region), with 95% confidence intervals.

**Table S-1:** Sample and Colorado population demographic comparison broken down by percentage.

|  | Sample Demographic  Percentages  n= 734 | Colorado Population Demographic  Percentages  n= 4,185,180 |
| --- | --- | --- |
| Regions |  |  |
| Front Range | 49.73% | 82.56 % |
| Western Slope | 37.74% | 14.12 % |
| Eastern Plains | 12.53% | 3.31 % |
| Age |  |  |
| 18-34 | 33.38 % | 32.05 % |
| 35-54 | 33.38 % | 34.72 % |
| 55+ | 32.97 % | 33.23 % |
| Gender |  |  |
| Male | 48.77% | 49.96 % |
| Female | 50.00% | 50.04 % |

*All population estimates were sourced from the American Community Survey 2017 5-Year Estimates

**Table S-2:** Sample sizes per geographic region and Colorado population estimates used for weighting data by geographic region

| Regions | Number in Sample or State Per Region | Percentage of Total Sample or State Population Per Region |
| --- | --- | --- |
| Sample Total | 734 |  |
| Sample Front Range | 365 | 49.73 |
| Sample Western Slope | 277 | 37.74 |
| Sample Eastern Plains | 92 | 12.53 |
| Colorado Total Population | 4,185,180 |  |
| Colorado Front Range Population | 3,455,465 | 82.56 |
| Colorado Western Slope Population | 591,030 | 14.12 |
| Colorado Eastern Slope Population | 138,685 | 3.31 |

*All population estimates were sourced from the American Community Survey 2017- 5 Year Estimates

**Table S-3:** Survey responses on measures related to intent to vote for or against wolf reintroduction, support for management options, and perceived personal impacts with comparison of unweighted proportions of responses and proportions weighted by state regional populations.

| **Measure** | **Sub-Group and Response Choice** | **Total in Sub-Group** | **Total in Sub-Group With Response Choice** | **Unweighted Proportion with Response Choice** | **Proportion with Response Choice Weighted by Region Populations** | **Weighted 95% Confidence Interval** |
| --- | --- | --- | --- | --- | --- | --- |
| Voting Intention Overall | Overall: Yes | 734 | 604 | 0.823 | 0.840 | [0.809, 0.872] |
| Voting Intention by Region | Western Slope:Yes | 277 | 221 | 0.798 | NA | [0.750, 0.845] (unweighted) |
|  | Front Range:Yes | 365 | 310 | 0.849 | NA | [0.812, 0.886] (unweighted) |
|  | Eastern Plains:Yes | 92 | 73 | 0.793 | NA | [0.709, 0.878] (unweighted) |
| Voting Intention by Community Size | Farm or Rural Area:Yes | 83 | 65 | 0.783 | 0.828 | [0.735, 0.922] |
|  | Town:Yes | 241 | 188 | 0.780 | 0.793 | [0.727, 0.860] |
|  | City:Yes | 410 | 351 | 0.856 | 0.852 | [0.814, 0.889] |
| Voting Intention by Children in Household | Children:Yes | 247 | 198 | 0.802 | 0.808 | [0.750, 0.867] |
|  | No Children:Yes | 486 | 405 | 0.833 | 0.857 | [0.820, 0.893] |
| Voting Intention by Pet Ownership | Own Cats or Dogs:Yes | 497 | 424 | 0.853 | 0.883 | [0.850, 0.915] |
|  | Do Not Own Cats or Dogs:Yes | 237 | 180 | 0.759 | 0.764 | [0.701, 0.827] |
| Voting Intention by Gender | Male:Yes | 358 | 300 | 0.838 | 0.854 | [0.813, 0.895] |
|  | Female:Yes | 367 | 296 | 0.807 | 0.821 | [0.772, 0.869] |
| Voting Intention by Age Group | 18-34:Yes | 229 | 189 | 0.825 | 0.833 | [0.773, 0.893] |
|  | 35-54:Yes | 262 | 223 | 0.851 | 0.864 | [0.817, 0.912] |
|  | 55+:Yes | 243 | 192 | 0.790 | 0.818 | [0.762, 0.874] |
| Voting Intention by Income | < 10k:Yes | 95 | 45 | 0.789 | 0.733 | [0.570, 0.897] |
|  | 10k-25k:Yes | 95 | 76 | 0.800 | 0.785 | [0.677, 0.893] |
|  | 25k-50k:Yes | 177 | 151 | 0.853 | 0.846 | [0.779, 0.913] |
|  | 50k-100k:Yes | 229 | 182 | 0.795 | 0.845 | [0.793, 0.897] |
|  | 100k-250k:Yes | 150 | 128 | 0.853 | 0.863 | [0.804, 0.923] |
|  | > 250k:Yes | 24 | 20 | 0.833 | 0.862 | [0.712, 1.00] |
| Voting Intention by Education | Less than High School:Yes | 19 | 12 | 0.632 | 0.653 | [0.359, 0.946] |
|  | High School:Yes | 211 | 171 | 0.810 | 0.819 | [0.753, 0.885] |
|  | Associate's:Yes | 162 | 142 | 0.877 | 0.894 | [0.839, 0.950] |
|  | Bachelor's:Yes | 204 | 163 | 0.799 | 0.816 | [0.756, 0.876] |
|  | Graduate/Professional:Yes | 138 | 116 | 0.841 | 0.858 | [0.793, 0.923] |
| Voting Intention by Identity | Wildlife Advocate:Yes | 513 | 451 | 0.879 | 0.894 | [0.863, 0.925] |
|  | Not Wildlife Advocate:Yes | 221 | 153 | 0.692 | 0.705 | [0.631, 0.778] |
|  | Animal Rights Advocate:Yes | 479 | 424 | 0.885 | 0.904 | [0.874, 0.934] |
|  | Not Animal Rights Advocate:Yes | 254 | 179 | 0.705 | 0.708 | [0.640, 0.777] |
|  | Gun Rights Advocate:Yes | 402 | 322 | 0.801 | 0.837 | [0.795, 0.879] |
|  | Not Gun Rights Advocate:Yes | 332 | 282 | 0.849 | 0.844 | [0.797, 0.890] |
|  | Property Rights Advocate:Yes | 508 | 418 | 0.823 | 0.842 | [0.805, 0.879] |
|  | Not Property Rights Advocate:Yes | 226 | 186 | 0.823 | 0.836 | [0.779, 0.894] |
|  | Hunter:Yes | 323 | 254 | 0.786 | 0.824 | [0.776, 0.873] |
|  | Identify Great Deal As Hunter:Yes | 78 | 48 | 0.615 | 0.661 | [0.530, 0.792] |
|  | Identify Moderate Amount As Hunter:Yes | 106 | 86 | 0.811 | 0.837 | [0.754, 0.920] |
|  | Identify Slight Amount As Hunter:Yes | 139 | 120 | 0.863 | 0.887 | [0.827, 0.948] |
|  | Not Hunter:Yes | 411 | 350 | 0.852 | 0.851 | [0.811, 0.892] |
|  | Rancher:Yes | 309 | 245 | 0.793 | 0.833 | [0.785, 0.881] |
|  | Identify Great Deal As Rancher:Yes | 74 | 48 | 0.649 | 0.695 | [0.567, 0.823] |
|  | Identify Moderate Amount As Rancher:Yes | 111 | 94 | 0.847 | 0.882 | [0.813, 0.950] |
|  | Identify Slight Amount As Rancher:Yes | 124 | 103 | 0.831 | 0.861 | [0.792, 0.930] |
|  | Not Rancher:Yes | 425 | 359 | 0.845 | 0.845 | [0.804, 0.887] |
|  | Conservationist:Yes | 507 | 431 | 0.850 | 0.876 | [0.843, 0.909] |
|  | Not Conservationist:Yes | 227 | 173 | 0.762 | 0.747 | [0.676, 0.818] |
| Support for Management Options | Limit for Deer Population Decline | 734 | 439 | 0.598 | 0.593 | [0.551, 0.636] |
|  | Compensate for Livestock Loss | 734 | 434 | 0.591 | 0.581 | [0.538, 0.624] |
|  | Compensate for Livestock Loss with Licensing Revenue | 734 | 417 | 0.568 | 0.580 | [0.537, 0.623] |
|  | Lethal Removal for Livestock Loss | 734 | 382 | 0.520 | 0.505 | [0.462, 0.549] |
|  | Recreational Hunting | 734 | 327 | 0.441 | 0.424 | [0.381, 0.466] |
|  | Compensate for Livestock Loss with Tax Revenue | 734 | 324 | 0.446 | 0.423 | [0.380, 0.466] |
| Perceived Personal Impact by Region | Western Slope:Negative Impact | 277 | 53 | 0.191 | NA | [0.145, 0.238] (unweighted) |
|  | Western Slope:No Impact | 277 | 133 | 0.480 | NA | [0.421, 0.539] (unweighted) |
|  | Western Slope:Positive Impact | 277 | 91 | 0.329 | NA | [0.273, 0.384] (unweighted) |
|  | Front Range:Negative Impact | 365 | 38 | 0.104 | NA | [0.073, 0.135] (unweighted) |
|  | Front Range:No Impact | 365 | 225 | 0.616 | NA | [0.566, 0.666] (unweighted) |
|  | Front Range:Positive Impact | 365 | 102 | 0.279 | NA | [0.233, 0.326] (unweighted) |
|  | Eastern Plains:Negative Impact | 92 | 11 | 0.120 | NA | [0.053, 0.186] (unweighted) |
|  | Eastern Plains:No Impact | 92 | 55 | 0.598 | NA | [0.497, 0.699] (unweighted) |
|  | Eastern Plains:Positive Impact | 92 | 26 | 0.283 | NA | [0.190, 0.375] (unweighted) |

**Table S-4:** Detailed results of qualitative coding of survey data and media analysis. Codes reported were mentioned by more than 3 participants.

| **Code** | **Example Quote(s)** | **% Survey Respondents (Who Indicated Wolves Would Impact Their Life, n= 320) Who Discussed Each Code** | **% of Media Articles (n=35) That Discussed Each Code** |
| --- | --- | --- | --- |
| ***Open-ended Responses to how would reintroduction would positively impact livelihoods or quality of life (n=216)*** | | | |
| ***Balance Ecosystem:*** Wolves would restore balance to ecosystems, return ecosystems to prior state, and/or enhance ecosystem/environment health | “It would make everything healthier by having the wolves back in Colorado.”  “Bringing wolves back into the ecosystem of Colorado would result in a healthier balance to nature.” | 19.38% | 54.29% |
| ***Observing Wolves:*** Would like to observe or listen to wolves in the wild; would increase participation in outdoor recreation if wolves were present | “I would try to see them in the wild, which would result in me getting out into nature more. Being [in] nature has been shown to have huge health benefits.”  “[I’d] be much more interested in hiking.”  “I think wolves could positively impact my livelihood and quality of my life because it could inspire me to be out in nature more.” | 15.63% | 11.43% |
| ***Emotional Connection:*** Loving wolves (or animals more generally), believing wolves are beautiful or majestic, or feeling an emotional or cultural connection to them | “My spouse and son love wolves and their happiness would make me happy.”  “Wolves are part of my culture so I feel a bond and close relationship to them.”  “Wolves have been a favorite animal of mine since childhood.”  “Wolves are majestic creatures.” | 10.31% | 5.71% |
| ***Moral arguments:*** Wolf reintroduction is the right thing to do; it makes up for past wrongs; wolves deserve to exist; humans should share our space with other beings | “We wouldn’t be the cause of their down fall anymore.”  “Because they were here first. We are the intruders.”  “It is always good to preserve species.”  “I think animals should be in their natural environment, not displaced for human greed.”  “Any move to [...] reverse some of the damage humans have caused is a positive thing for all humans (including myself)”    “Living in Colorado you have to share your space, as the wildlife shares theirs with people”  “Would bring a great feeling that we are righting a wrong” | 9.38% | 8.57% |
| ***Control Pests:*** Wolves would control pest populations that affect people (e.g. coyotes, deer, rodents) | “It would reduce the rodent populations”  “Wolves would help control rabbit, coyote, and other wildlife populations naturally.”  “Decrease chance of hitting deer/elk on roadways”  “The deer population is out of control in my opinion and having a natural predator to curtail that population would be a good thing” | 6.88% | 0.00% |
| ***Existence Value:***  Knowing wolves exist now or for future generations and/or the ecosystem is intact would increase happiness and satisfaction | “Because my child would grow up knowing wolves existed.”  “Knowing that wildlife is comfortably thriving around me”  “Knowing they are possibly going to be reintroduced into Colorado gives me positive feelings.”  “I would like to know that wildlife can still live and thrive where they belong”  “It would feel good to have them a part of Colorado.” | 4.38% | 8.57% |
| ***Tourism Revenue:***  Wolf reintroduction would increase tourism opportunities | “It would increase tourism”  “Wolves would increase tourism and help with jobs” | 2.19% | 0% |
| ***Learning and Environmental Awareness:***  Wolf reintroduction would increase environmental learning and care | “Cause more attention to protecting them and their habitats”  “I believe wolves would help me learn more about them when seeing them in nature. Wolves are different in captivity.”  “It would make me appreciate the wilderness even more”  “Heighten my appreciation of wilderness knowing there are wolves there” | 1.88% | 2.86% |
| ***Increase Wildlife Diversity:***  Wolf reintroduction would increase the diversity or abundance of wildlife in Colorado | “It’s always wonderful to have more wildlife around you”  “Increase the variety of wildlife”  “It is important they contribute to the biodiversity of this area”  “There would be more native animals around.” | 1.88% | 8.57% |
| ***Reduction in Ungulate Disease/Sickness and Provisioning of Other Ecosystem Services:***  Wolf reintroduction would reduce the number of sick ungulates or ungulate disease and provide other services for humans | “Currently there is a large population of mule deer that wander the neighborhood of Colorado Springs. Introducing wolves could help control this population reducing car accidents and deer wasting disease”  “We would seldom see sick or dead animals that were the wolves prey out in nature”  “Help remove sick animals that could spread disease to humans” | 1.88% | 8.57% |
| ***CO State Pride:*** Wolf reintroduction would enhance Colorado pride and/or make Colorado better | “It would put Colorado in good news for being responsible to correcting an environmental wrong”  “It would increase the wildness of Colorado.”  “It would make me feel better about the place I live.”  “It would be cool to be in a state that supports their reintroduction.” | 1.56% | 11.43% |
| ***Open-ended Responses to how wolves would negatively impact livelihoods or quality of life (n=104)*** | | | |
| ***Human Safety:***  Fear of wolves posing a threat to human safety and/or wandering into residential areas and causing harm | “I would be afraid to walk around outside. I would be afraid wolves would get into trash cans, backyards, public areas. I would be afraid a wolf would bite or attack me.”  “As they get established they will multiply and move into residential areas, just as bears, mountain lions, etc, have done.”  “I would be slightly more afraid of being in wilderness areas.”  “I would stop doing outdoor activities and would always be afraid of going outside.” | 19.06% | 25.71% |
| ***Hunting Opportunities:*** Reduction in hunting opportunities | “Because if might ruin my chance to fill my tag during hunting season.”  “Being a hunter there are just a few opportunities to hunt in Colorado, and wolves would greatly reduce that.”  “Hunting is a big part of my family. If having wolves made it harder to get tags for hunting it might push us to move.”  “I hunt deer and elk for food for my family. If they are reduced in numbers it will be harder to feed my family.” | 5.00% | 14.29% |
| ***Pet Attacks:*** Fear of wolves posing a threat to pets | “We live in the mountains at 7300 feet and would be cautious about walking our gravel road with our little dog.”  “I would worry about my pets being outside.”  “I have 2 dogs that I walk every day near a Colorado Wildlife Area. I am extremely concerned about coyotes, snakes, and mountain lions. Adding wolves to that population doesn’t thrill me.” | 4.06% | 11.43% |
| ***Livestock Loss:***  Concerns about depredation on livestock and threats to ranching income | “We are a farming family, live on a farm, main source of income comes from farming [...] All of these things are impacted by a wolf population.”  “Many of the local ranches would be impacted by wolves being reintroduced to Colorado. My family buy local meat, which could be affected.”  “They would attack livestock.” | 3.75% | 51.43% |
| ***Reduce Wildlife:*** Wolves would reduce diversity and abundance of wildlife, namely deer and elk. | “I feel like wolves will make a decrease the population of other animals and can possibly harm humans too”  “me and my father like to hunt deer and elk, and I believe the elk and deer population would decrease or relocate” | 2.19% | 45.71% |
| ***Wolves are “Killers”:***  Concern that wolves are killers/predators/cruel animals | “They hunt and kill.”  “Knowing these animals that attack and kill for pleasure and not for surviving.”  “Because they are mean.” | 1.88% | 8.57% |
| ***Compensation/Management:*** Wolves will be poorly managed or difficult to manage | “I have seen this first hand in Wyoming, Idaho, and Montana. The government stopped paying for wolf killed livestock. They claim they will pay but make it impossible to get compensation.”  “The government will not pay for livestock because they say “it could have killed by something else”  “States have had nothing but trouble with wolves and have even had to permit wolf hunts to help control the size of the wolf packs.” | 1.56% | 22.86% |

# *Survey Instrument*

Below are copies of the survey questions discussed in this paper.

To what extent do you feel that wolves would have a direct impact on your livelihood or quality of life?

- They would have a strong negative impact (1)
- They would have a moderate negative impact (2)
- They would have a slight negative impact (3)
- They wouldn't have an impact (4)
- They would have a slight positive impact (5)
- They would have a moderate positive impact (6)
- They would have a strong positive impact (7)

Briefly describe why you feel wolves would (negatively/positively) impact your livelihood or quality of life

________________________________________________________________

________________________________________________________________

________________________________________________________________

________________________________________________________________

________________________________________________________________

If wolf reintroduction were to occur and wolves became reestablished in Colorado, is it acceptable or unacceptable in the future for wildlife management agencies to....

|  | Highly unacceptable (1) | Moderately unacceptable (2) | Slightly unacceptable (3) | Neither (4) | Slightly acceptable (5) | Moderately acceptable (6) | Highly acceptable (7) |
| --- | --- | --- | --- | --- | --- | --- | --- |
| Limit the number of wolves if they cause declines in deer and elk populations in certain areas? (1) | ○ | ○ | ○ | ○ | ○ | ○ | ○ |
| Capture and lethally remove a wolf if it is known to have caused loss of livestock? (2) | ○ | ○ | ○ | ○ | ○ | ○ | ○ |
| Compensate landowners for loss of livestock caused by a wolf? (3) | ○ | ○ | ○ | ○ | ○ | ○ | ○ |
| Use a portion of state hunting and fishing license dollars to compensate landowners for loss of livestock caused by a wolf? (4) | ○ | ○ | ○ | ○ | ○ | ○ | ○ |
| Use a portion of state tax dollars to compensate landowners for loss of livestock caused by a wolf? (5) | ○ | ○ | ○ | ○ | ○ | ○ | ○ |
| Allow a recreational hunt of wolves once they have reached a certain population size that exceeds recovery goals? (6) | ○ | ○ | ○ | ○ | ○ | ○ | ○ |

If you were given the opportunity to vote for or against reintroducing the gray wolf into Colorado, how would you vote?

- I would vote for reintroducing the gray wolf (1)
- I would vote against reintroducing the gray wolf (0)

Please indicate the extent to which you identify yourself as a/an... (Please select one for each)

|  | Do not identify with group at all (1) | Identify with group a slight amount (2) | Identify with group a moderate amount (3) | Identify with group a great deal (4) |
| --- | --- | --- | --- | --- |
| Wildlife advocate (1) | ○ | ○ | ○ | ○ |
| Animal rights advocate (2) | ○ | ○ | ○ | ○ |
| Gun rights advocate (3) | ○ | ○ | ○ | ○ |
| Property rights advocate (4) | ○ | ○ | ○ | ○ |
| Hunter (5) | ○ | ○ | ○ | ○ |
| Rancher (6) | ○ | ○ | ○ | ○ |
| Conservationist (7) | ○ | ○ | ○ | ○ |

Are you....?

- Male (1)
- Female (2)
- Non-binary/third gender (3)
- Prefer to self-describe (3)
- Prefer not to say (3)

How many people under 18 years of age are currently living in your household?

________________________________________________________________

Do you have any pets in your household? (Select all that apply)

- Dog (1)
- Cat (2)
- Other type of pet(s) (3) ________________________________________________
- No pet (4)

What is your annual household income before taxes? (Select one)

- Less than $10,000 (1)
- $10,000 to less than $25,000 (2)
- $25,000 to less than $50,000 (3)
- $50,000 to less than $100,000 (4)
- $100,000 to less than $250,000 (5)
- $250,000 or more (6)

What is the highest level of education you have completed? (Select one)

- Less than high school (1)
- High school diploma or equivalent (e.g., GED) (2)
- 2-year associate's degree or trade school (3)
- 4-year college degree (4)
- Advanced degree beyond 4-year college degree (5)

How would you describe your current residence or community? (Select one)

- Large city with 250,000 or more people (1)
- City with 100,000 to 249,999 people (2)
- City with 50,000 to 99,999 people (3)
- Small city with 25,000 to 49,999 people (4)
- Town with 10,000 to 24,999 people (5)
- Town with 5,000 to 9,999 people (6)
- Small town or village with less than 5,000 people (7)
- A farm or rural area (8)
